# Longitudinal Transitions Across Physical Function States Among Older Cancer Survivors

**DOI:** 10.1093/geroni/igaf122.269

**Published:** 2025-12-31

**Authors:** Emilie Duchesneau, Allison Musty, Kathryn Callahan, Heidi Klepin, Amresh Hanchate, Mara Vitolins, Nicholas Pajewski

**Affiliations:** Wake Forest University School of Medicine, Winston-Salem, North Carolina, United States; Wake Forest University School of Medicine, Winston-Salem, North Carolina, United States; Wake Forest University School of Medicine, Winston-Salem, North Carolina, United States; Wake Forest University School of Medicine, Winston-Salem, North Carolina, United States; Wake Forest University School of Medicine, Winston-Salem, North Carolina, United States; Wake Forest University School of Medicine, Winston-Salem, North Carolina, United States; Wake Forest University School of Medicine, Winston-Salem, North Carolina, United States

## Abstract

Older cancer survivors are at risk of functional decline. Changes in physical function in older cancer survivors are understudied. We described longitudinal transitions in physical function among older cancer survivors using Rounds 1-12 (2011-2022) of the National Health and Aging Trends Study, a nationally representative cohort of adults aged 65+ years. We included participants with a self-reported cancer history in Round 1. Physical function was assessed annually based on limitations (unable to do without assistance) in 4 self-care, 3 mobility, and 5 household activity domains and was categorized as no limitations (0), minimal limitations (1-3), or moderate-to-severe limitations (4-12). Multistate Markov models estimated 1-year transition probabilities across physical function states and death, pooling across rounds. Among 1303 cancer survivors (56.9% female, 46.3% aged 65-74, 38.3% aged 75-84, 15.4% aged 85+), 42.2% had minimal and 32.2% had moderate-to-severe limitations at baseline. Individuals without limitations had a 62% probability of remaining stable, 32% probability of developing minimal limitations, 5% probability of developing moderate-to-severe limitations, and 2% probability of dying over one year. Those with minimal limitations had a 66% probability of maintaining their status, a 10% probability of improving to no limitations, a 19% probability of progressing to moderate-to-severe limitations, and a 5% probability of dying. In those with moderate-to-severe limitations, the probabilities of remaining in the state, improving, and dying were 72%, 13%, and 14%, respectively. These results highlight the dynamic nature of physical function in older cancer survivors and suggest that transitions to greater limitations are more common than recovery.

